# IL-13 May Could Enhance the Proliferation and Affect the Differentiation of Nasal Epithelium Basal Cells Through the mTOR/p70S6K1 Pathway in Chronic Rhinosinusitis With Nasal Polyps

**DOI:** 10.1155/mi/8108993

**Published:** 2025-05-21

**Authors:** Ping Li, Tao Li, Jinfeng Luo, Peng Yu, Tao Jiang, Xiangmin Zhou, Liang Yu, Aiping Chen, Yuzhu Wan, Li Shi

**Affiliations:** ^1^Department of Otolaryngology-Head and Neck Surgery, Shandong Provincial ENT Hospital, Shandong University, Jinan, China; ^2^Department of Allergy, Shandong Provincial ENT Hospital, Shandong University, Jinan, China; ^3^Department of Rhino-Inflammatory Disease, Shandong Provincial ENT Hospital, Shandong University, Jinan, China; ^4^Shandong Provincial Key Medical and Health Laboratory of Airway Inflammatory Disease, Jinan, China; ^5^Department of Nasal-Skull Base Oncology, Shandong Provincial ENT Hospital, Shandong University, Jinan, China; ^6^Sturctral Rhinology Department, Shandong Provincial ENT Hospital, Shandong University, Jinan, China; ^7^Department of Otolaryngology, The Second Hospital of Shandong University, Jinan, China

**Keywords:** CRSwNP, differentiation, mTOR, p70S6K1, proliferation

## Abstract

**Background:** One of the hallmarks of Chronic rhinosinusitis with nasal polyps (CRSwNP) is the overexpression of IL-13, which may influence the proliferation and differentiation of nasal epithelial basal cells. However, the pathway is not clear enough, and the mTOR/p70S6K1 pathway is related to cell growth. This study was trying to explore if IL-13 could impact nasal epithelial basal cells through the mTOR/p70S6K1 pathway.

**Methods:** PCR, western blot (WB), and immunohistochemistry (IHC) were used to compare the difference between IL-13 and the mTOR/p70S6K1 pathway-related molecules expression level between the healthy control (HC) and CRSwNP groups. WB, 5-ethynyl-2′-deoxyuridine staining, and Immunofluorescent (IF) were performed on human nasal epithelial progenitor cells (HNEPCs) to detect the proliferation ability under the effect of IL-13. In addition, qRT-PCR, WB, and IF were used to detect the differentiation ability with the stimulation of IL-13 in the air-liquid interface (ALI) system.

**Results:** The expression of IL-13, mTOR/p70S6K1-related molecules, and proliferation-related molecules Ki67, CDK2, and cyclin E1 were upregulated in CRSwNP compared to HC. In HNEPCs, IL-13 could stimulate nasal epithelial cells proliferating through the mTOR/p70S6K1 pathway, and this phenomenon could be inhibited when mTOR (with rapamycin) and S6K1 (with PF-4708671) were blocked. In the ALI system, the effect of IL-13 added in the proliferation phase could persist in the proliferation and differentiation stage, affecting the nasal epithelial progenitor/stem cells' irregular differentiation.

**Conclusion:** IL-13 may affect the proliferation and differentiation of nasal epithelial progenitor/stem cells through the mTOR/p70S6K1 pathway, which may affect the development of nasal polyps.

## 1. Background

Chronic rhinosinusitis with nasal polyps (CRSwNP) is one kind of chronic inflammatory disease with nasal polyposis, affecting about 2.5% of population around the world [1]. CRSwNP is highly heterogeneous and can be classified into three endotypes based on the mainly expressed Th1, 2, and 3 cytokines, respectively [2]. Type 2 immune response takes the most part endotype of CRSwNP [3], which has severe clinical symptoms and a higher rate of recurrence [1, 4]. T2 inflammation was caused by Th2 cells, group 2 innate lymphoid cells (ILC2s), and mast cells, producing IL-4, 5, 13, and other inflammatory cytokines, leading to nasal epithelial barrier dysfunction, tissue remodeling, IgE response enhancement, and eosinophil infiltration [2, 5, 6]. Nasal epithelial cells include basal, ciliated, columnar, and goblet cells [7]. Tissue remodeling includes abnormal epithelial proliferation, fibrosis, basal membrane thickness, goblet cell hyperplasia, and mucus irregular secretion [8]. Basal cells of epithelium have the potential for multidirectional differentiation, which could be differentiating into different types of epithelial cells, including cilia cells and goblet cells [9]. Hyperproliferation of nasal epithelium during tissue remodeling is mainly manifested by abnormal proliferation of basal cells. In our previous study, we confirmed that P63 can serve as a marker for nasal basal cells. And we observed that in nasal polyp tissues, the expression of P63 was elevated in areas of squamous metaplasia, and a multilayer of p63-positive basal cells with areas of squamous metaplasia, its tissue localization was altered. Thus, regardless of whether basal cells undergo abnormal proliferation, P63 can serve as a marker for nasal basal cells [10].

Studies declared that the expression of IL-13 in CRSwNP is significantly upregulated compared with healthy control (HC) [11, 12]. The expression level of Ki67, a classical proliferation marker, is also evaluated in CRSwNP [13]. Some researchers indicated that IL-13 may enhance airway goblet cell hyperplasia and smooth muscle cell proliferation [14], and this function could be observed in many other types of cells, such as mammary epithelial cells, gastrointestinal tumor cells, and conjunctival goblet cells [15–17]. However, the specific mechanism of IL-13 promoting the proliferation in nasal epithelium cell has yet to be identified.

The PI3K/mTOR pathway plays a vital role in many physiological processes such as cell growth, glucose homeostasis, adipocyte metabolism, and energy balance [18, 19]. The mammalian target of rapamycin (mTOR) is a serine/threonine protein kinase in mammals. When mTOR is activated, it will phosphorylate to promote cell anabolism and inhibit catabolism, but rapamycin could inhibit the activation of mTOR, reducing the downstream molecular activation of 70 kDa ribosomal protein S6 kinase 1 (p70S6K1), a direct substrate downstream of mTOR, which also plays its physiological role through phosphorylation. p70S6K1 plays an essential role in cell proliferation and cell cycle transition by promoting G1/S phase transition [20]. Cyclin-dependent kinase 2 (CDK2) can bind cyclin E1 in the G1 phase to accelerate the cell cycle to S phase, which are the related factors that affect G1/S phase transition [21, 22].

This study aimed to investigate whether IL-13 could influence the expression and function of the mTOR/p70S6K1 signaling pathway to promote the proliferation in nasal epithelium cell, and in addition, to verify whether IL-13 could affect the differentiation of nasal epithelial progenitor/stem cells.

## 2. Materials and Methods

### 2.1. Patients and Samples

CRSwNP and HC patients were all collected from the Department of Otolaryngology of Shandong Provincial ENT Hospital, China. 67 CRSwNP biopsies were obtained from endoscopic sinus surgery (ESS), and 29 HC samples were taken from the middle turbinate mucosa of patients with nasal septum deviation and bullous middle turbinate under septal plastic surgery and middle turbinate surgery. The diagnostic standards of CRSwNP were according to the European Position Paper on Rhinosinusitis and Nasal Polyps 2020 (EPOS2020) [1], the patients of CRSwNP and HC had no upper airway infection and were not on any corticosteroid medications within 3 months before surgery. Written consents from all patients were obtained and the local ethical committee (number: XYK202111008).

Patient's clinical characteristics are shown in Table 1. Fresh specimens were separated into three parts: one portion stored at −80 °C for protein extraction, one portion preserved with RNA later (Invitrogen, Carlsbad, CA, USA) stored at −80 °C for RNA extraction, and one portion fixed in formalin for histological staining.

### 2.2. Human Nasal Epithelial Stem/Progenitor Cells (HNESPCs) Culture

**Figure 1:**
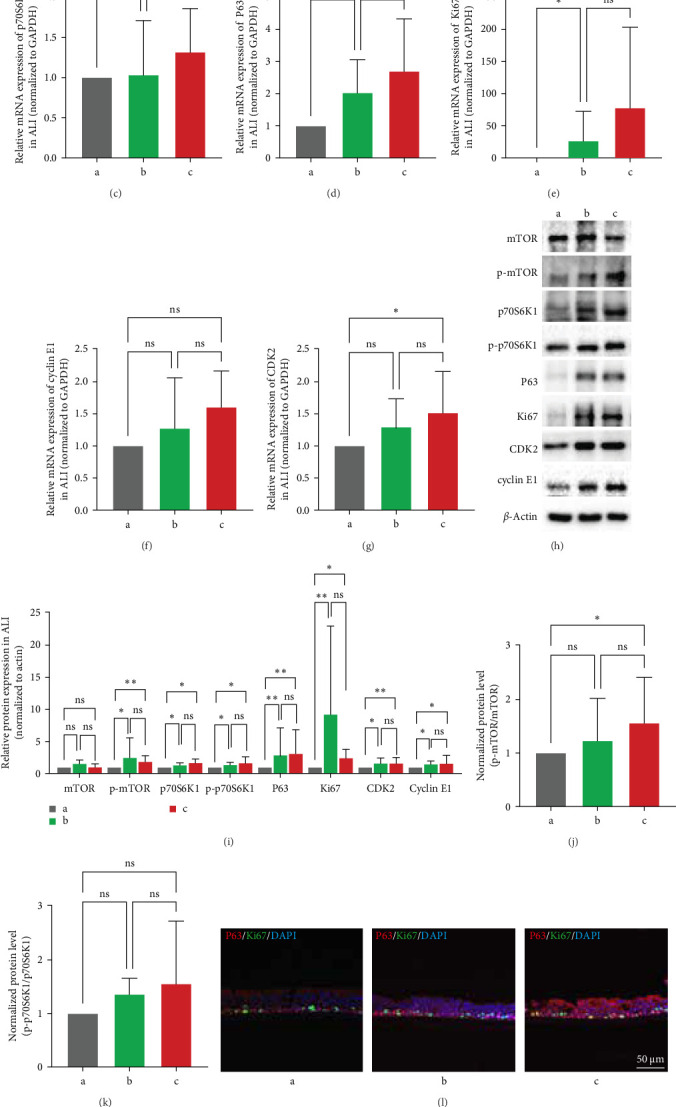
The effect of IL-13 stimulated in different phases in the ALI system. (A) Study protocols, *n* (a, b, c) = 5. (B–G) Relative mRNA expression level of mTOR, p70S6K1, P63, Ki67, CDK2, and cyclin E1 were detected by RT-PCR. (H) and (I) representative images of western blot analysis and relative protein expression level of mTOR, p-mTOR, p70S6K1, p-p70S6K1, P63, Ki67, CDK2, and cyclin E1. (J) and K) show the normalized protein level of p-mTOR/mTOR and p-p70S6K1/p70S6K1. (L–O) Represented images of Ki67, P63 and double IF staining in ALI system were photographed with the confocal laser scanning microscope and its positive cell number, 400x magnification, and scale bar = 50 µm. Data were shown as mean ± SD, statistical significance was analyzed by one-way ANOVA test. ALI, air-liquid interface; *⁣*^*∗*^*p* < 0.05; *⁣*^*∗∗*^*p* < 0.01; *⁣*^*∗∗∗*^*p* < 0.001; ns, no significance.

HNESPCs were isolated from fresh HC samples, which were cultured as previously reported [23]. The relevant identification of HNESPCs was shown in Figure S4. For part one, after HNEPCs adherence, the culture medium was replaced with fresh medium containing Recombinant Human IL-13 (10 ng/ml, Peprotech, Rocky Hill, NJ, USA), and the cells were incubated for 48 h. Before that, HNEPCs were divided into four groups: (1) cells were pretreated with DMSO (0.1%) as the vehicle control for 1 h; (2) cells were treated with IL-13 (10 ng/ml) only; (3) cells were pretreated with rapamycin (10 nM, Selleck, Houston, Texas, USA), a specific mTOR inhibitor, for 1 h; (4) cells were pretreated with PF-4708671 (10 nM, Selleck, Houston, Texas, USA), a specific p70S6K1 inhibitor, for 1 h. For part two, when HNEPCs reached a density of 70%, we transplanted the cells into transwell membranes to construct an air-liquid interface (ALI) system to form a pseudostratified layer within 35 days. The ALI system included two phases of epithelium: the duration of proliferation for the first 5 days after cell adhesion and the duration of differentiation for the last 28 days. Divided the ALI system into three groups (Figure 1A): (a) added PBS as vehicle control; (b) added IL-13 (10 ng/ml) during the duration of proliferation; (c) added IL-13 (10 ng/ml) during the entire duration of proliferation and differentiation. In addition, we transferred the portion of cells and pretreated them with inhibitors for 1 h before IL-13 (10 ng/ml) was added at the proliferation phase. Cells were pretreated with rapamycin (10 nM) and PF-4708671 (10 nM), and DMSO was used as the vehicle control.

### 2.3. RNA Extraction and Reverse Transcription-Polymerase Chain Reaction (RT-PCR)

Total RNA was extracted with TRIzol reagent (Invitrogen, Carlsbad, CA, USA). Then 1 μg of total RNA was reverse transcribed into cDNA using PrimeScript RT reagent Kit with gDNA Eraser (TaKaRa, Kusatsu, Shiga, Japan) following manufacturer's protocol. The polymerase chain reaction procedures for the amplification were conducted in the following order: 30 s at 95 °C for denaturation, 5 s at 95 °C, and 31 s at 60 °C for 40 cycles, dissociation for 15 min. The housekeeping gene was GAPDH, and the relative gene expression was calculated by the comparative 2^−*△△*CT^ method, normalized against the housekeeping gene. The primer sequences are shown in Table 2.

### 2.4. Western Blot (WB) Analysis

Frozen nasal tissues and cells were homogenized in RIPA Lysis Buffer (Beyotime, Shanghai, China) containing 1% PMSF (Solarbio, Beijing, China) and 1% phosphatase inhibitor (MCE, Shanghai, China). After centrifugation at 4 °C for 15 min, the supernatant was collected, and the protein concentration was measured by BCA method. Each lane was added 30 μg protein with 8% or 10% SDS-PAGE and transferred to PVDF membranes. The membranes were blocked with 5% skim milk for 1 h and incubated with primary antibodies (Table S1) overnight at 4 °C. After that, the membranes were incubated with appropriate secondary antibodies for 1 h at RT. The membranes were detected by ECT reagent (Bio-Rad, California, USA). Image J was used to analyze the blot bands.

### 2.5. Immunohistochemistry (IHC) and Immunofluorescent (IF)

Tissues and transwell membranes of ALI cultures were embedded in paraffin and sectioned at 4 μm with a Leica microtome (Leica, Wetzlar, Germany). After deparaffinized and hydrated, the sections were heated for 25 min at 95 °C with pH6/9 retrieval buffer (Abcam, Cambridge, CB2 0AX, UK) for antigen retrieval.

Tissue sections were performed with IHC staining, and endogenous peroxidase activity was blocked with 3% H_2_O_2_ for 15 min at RT. Then the sections were blocked with 10% goat serum for 30 min at RT and incubated with primary antibodies (Table S1) at 4 °C overnight. The sections were then incubated with GTVision + detection system-HRP (Gene Tech, Shanghai, China) for 30 min at RT, after which diaminobenzidine substrate was added for color development. All sections were counterstained with hematoxylin. All IHC staining sections were photographed with a light microscope (Olympus BX53, Tokyo, Japan). The measurement parameters in IHC were to calculate the mean optical density values (Integrated optical density/Area, IOD/Area) of the positive cells that showed brown articles or clumps in the cytoplasm within the epithelium, and in IF staining, the KI67 and P63 were to count the positive cell number in the cell nucleus within the epithelium. The positive staining areas in the HCs and NPs were captured under 400x amplification and were assessed by using Image-Pro Plus (five measurements per section).

Double IF staining of cytospin samples and transwell membranes was performed with two different host species antibodies: P63 and Ki67, MUC5AC, and *β*-Tublin. The slides were permeabilized with 0.2% Triton X-100 (Solarbio, Beijing, China) for 10 min at RT and blocked with 10% goat serum for 30 min. After that, the slides were incubated with primary antibodies (Table S1) at 4 °C overnight and followed by Alexa Fluor 488 and 594 conjugated secondary antibodies (Invitrogen, Carlsbad, CA, USA) at 1:500 for 1 h incubation in the dark at RT and followed by mounting the slides with Antifade reagent with DAPI (Invitrogen, Carlsbad, CA, USA). Cytospin slides were analyzed with fluorescence microscopy (Olympus BX53, Tokyo, Japan) and transwell membranes with a confocal laser scanning microscope (Leica SP8, Wetzlar, Germany) under 400x amplification (five measurements per section). The expression of Ki67 and P63 was used to calculate the positive cell number within the epithelium or the positive cell ratio and assessed by using Image-Pro Plus.

### 2.6. Statistical Analysis

SPSS 26.0 (SPSS Inc., Chicago, IL, USA) and GraphPad Prism 9.0.8 (GraphPad Software, La Jolla, CA, United States) were used to analyze the data. The Shapiro–Wilk test was used to detect the normality of the data. Variables were described using mean ± standard deviation (SD). Student's *t*-tests were performed to determine the statistical significance between the two groups. The one-way ANOVA test was used for more than two groups, respectively. All in vitro experiments were done and repeated at least three times. Correlation analysis was performed using Pearson correlation. In all analyses, *p* < 0.05 was considered statistically significant. All the *p*-values were listed on Table S2–S5.

## 3. Results

1. The expression of mTOR/p70S6K1 pathway and proliferation-related molecules was upregulated in CRSwNP compared to HC.

**Figure 2:**
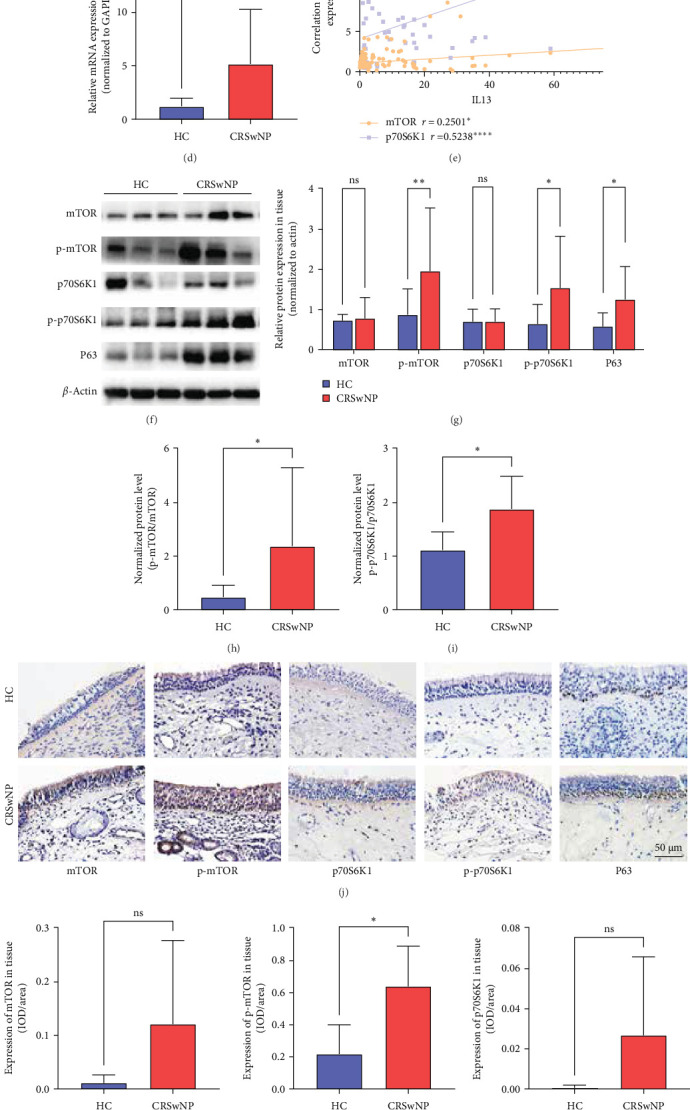
The expression level of mTOR/p70S6K1 pathway-related molecules and P63 in HC and CRSwNP. (A–D) Relative mRNA expression level of IL-13, mTOR, p70S6K1, P63 in HC and CRSwNP were detected by RT-PCR, *n* (HC) = 29, n (CRSwNP) = 67. (E) The positive correlation of IL-13 with mTOR (*r* = 0.2501) and IL-13 with p70S6K1 (*r* = 0.5238) were recorded at the mRNA level by Spearman correlation. (F) and (G) representative images of western blot analysis and relative protein expression level of mTOR, p-mTOR, p70S6K1, p-p70S6K1, P63 in HC and CRSwNP, n (HC) = 12, n (CRSwNP) = 12. (H) and (I) show the normalized protein level of p-mTOR/mTOR and p-p70S6K1/p70S6K1. (J) and (K) representative images and semiquantitative analysis of mTOR, p-mTOR, p70S6K1, p-p70S6K1, P63 in immunochemistry staining was quantified by Image Pro Plus software according to the IOD to area ratio, *n* (HC) = 12, *n* (CRSwNP) = 12, 400x magnification, and scale bar = 50 µm. Data were shown as mean ± SD, statistical significance was analyzed by student's *t* test. HC, healthy control; CRSwNP, chronic rhinosinusitis with nasal polyps; IOD, integrated optical density; *⁣*^*∗*^*p* < 0.05; *⁣*^*∗∗*^*p* < 0.01; *⁣*^*∗∗∗*^*p* < 0.001; *⁣*^*∗∗∗∗*^*p* < 0.0001; ns, no significance.

Expression level of IL-13, P63, and mTOR/p70S6K1 pathway-related-molecule expression levels in CRSwNP and HC were first evaluated by qRT-PCR, WB, and IHC. First, the qRT-PCR indicated that mRNA production of IL-13, P63, mTOR, and p70S6K1 was upregulated in CRSwNP compared to HC (Figure 2A–D, *p* < 0.01). Then correlations between IL-13 and mTOR, p70S6K1 were analyzed by GraphPad Prism 9.0.8, showing that IL-13 positively correlated with mTOR (Figure 2E, *r* = 0.2501, *p* < 0.05) and p70S6K1 (Figure 2 = 0.5238, *p* < 0.01). Second, the WB results indicated that protein level of p-mTOR, p-p70S6K1, and P63 were significantly increased in CRSwNP compared to HC (Figure 2F,G, *p* < 0.05). mTOR and p70S6K1 production in CRSwNP also have an upregulated trend, but there were no statistical differences (Figure 2F,G, *p* > 0.05). Figure 2H,I intuitively demonstrates that, compared with NC, the expression levels of p-mTOR and p-p70S6K1 in CRSwNP are significantly elevated. Last, the IHC staining results reminded that p-mTOR and p-p70S6K1 relative expression in basal cells were upregulated (Figure 2I,J, *p* < 0.05), having the same results as WB. mTOR production in CRSwNP epithelium basal cells also increased, but the statistical analysis indicated that the *p*-value was larger than 0.05. P63, as a nasal epithelial basal cell-specific marker, its relative expression was increased in CRSwNP (Figure 2J,K, *p* < 0.05), meaning that the basal cells proliferation in nasal epithelium was enhanced. Therefore, we hypothesize that IL-13 may stimulate mTOR and p70S6K1 expression to influence the proliferation of basal cells in nasal epithelium.

**Figure 3:**
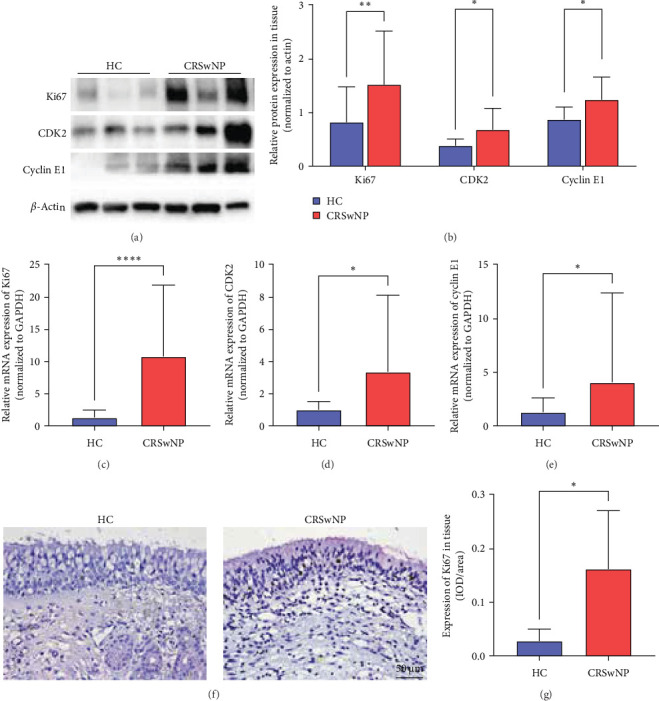
Ki67, CDK2, and cyclin E1 expression levels in CRSwNP and HC. (A) and (B) representative images of western blot analysis and relative protein expression level of Ki67, CDK2, and cyclin E1 in CRSwNP and HC, *n* (HC) = 12, *n* (CRSwNP) = 12. (C–E) Relative mRNA expression level of Ki67, CDK2, and cyclin E1 were detected by RT-PCR, *n* (HC) = 29, *n* (NP) = 67. (F) and (G) Ki67 representative images and semi-quantitative analysis of mean optical density value (IOD/area) in immunochemistry staining was quantified by Image Pro Plus software, *n* (HC) = 12, *n* (CRSwNP) = 12, 400x magnification, and scale bar = 50 µm. Data were shown as mean ± SD, statistical significance was analyzed by student's *t* test. HC, healthy control; CRSwNP, chronic rhinosinusitis with nasal polyps; IOD, Integrated optical density; *⁣*^*∗*^*p* < 0.05; *⁣*^*∗∗*^*p* < 0.01; *⁣*^*∗∗∗∗*^*p* < 0.0001.

To verify if IL-13 could influence the proliferation of nasal epithelial basal cells, WB, qRT-PCR, and IHC were performed to verify if proliferation-related molecules, such as Ki67, cell cycle-related factors cyclin-dependent kinase 2 (CDK2), and cyclin E1 were enhanced in CRSwNP. First, we detected the protein level of proliferation maker Ki67, CDK2, and cyclin E1 by WB, revealing that CRSwNP had much greater levels of these components than HC (Figure 3A,B, Ki67, *p* < 0.01; CDK2 and cyclin E1, *p* < 0.05). Then qRT-PCR was performed to demonstrate the mRNA level of Ki67, CDK2, and cyclin E1, the same as WB results, all those three factors were also upregulated in CRSwNP (Figure 3 Ki67, *p* < 0.0001; CDK2 and cyclinE1, *p* < 0.05). IHC staining also proved that mean optical density values of Ki67 were significantly higher in CRSwNP and mainly expressed in the nucleus of nasal epithelial basal cells (Figure 3F,G, *p* < 0.05). These data indicated that the proliferative activity of CRSwNP epithelium was higher than that of the HC group.

2. IL-13 could stimulate nasal epithelial cells' proliferation through mTOR/p70S6K1 pathway.

**Figure 4:**
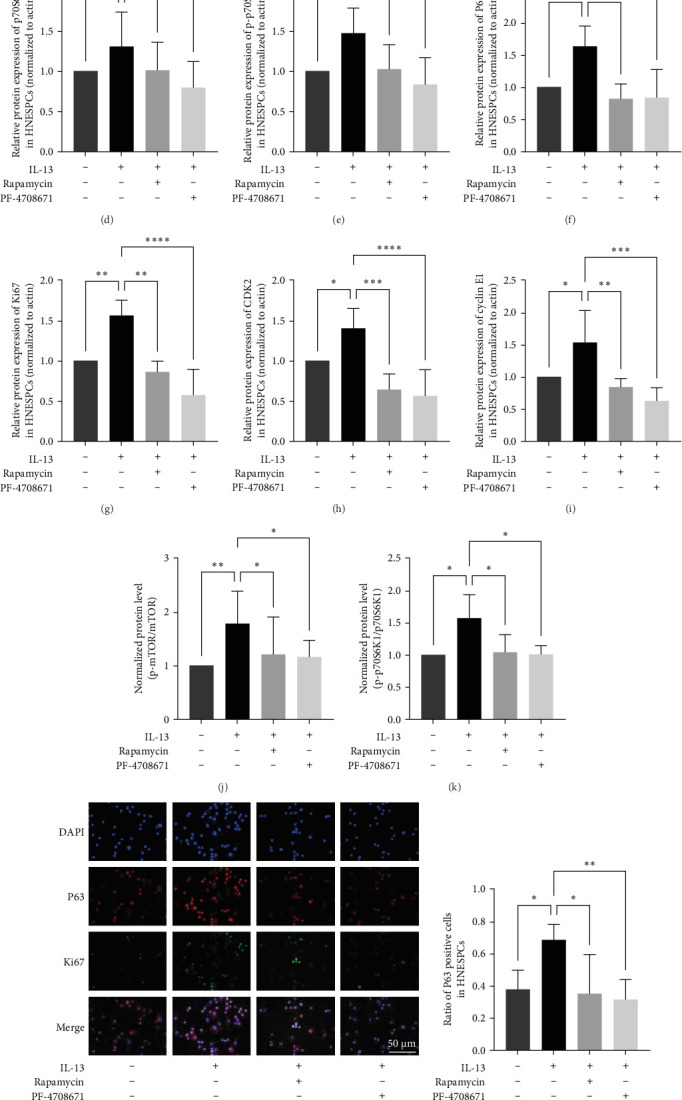
IL-13 stimulated mTOR/p70S6K1 pathway related molecules expression, rapamycin, and PF-4708671 reversed its impact in HNESPCs (*n* = 5). (A–E) and (F–I) representative images of western blot analysis and relative protein expression level of mTOR, p-mTOR, p70S6K1, p-p70S6K1, P63, Ki67, CDK2, and cyclin E1. (J) and (K) show the normalized protein level of p-mTOR/mTOR and p-p70S6K1/p70S6K1. (L–O) Represented images of Ki67, P63, and double IF staining were photographed with fluorescence microscopy, 400x magnification, and scale bar = 50 µm. Data were shown as mean ± SD, statistical significance was analyzed by one-way ANOVA test. HNESPCs, human nasal epithelial stem/progenitor cells; *⁣*^*∗*^*p* < 0.05; *⁣*^*∗∗*^*p* < 0.01; *⁣*^*∗∗∗*^*p* < 0.001; *⁣*^*∗∗∗∗*^*p* < 0.0001; ns, no significance.

To verify the proliferative effects of IL-13 and its impact on mTOR/p70S6K1 signaling pathway, we stimulated HNEPCs with human recombinant IL-13 (10 ng/ml), mTOR inhibitor rapamycin (10 nM), and S6K1 inhibitor PF-4708671 (10 nM) as previously described. After 48 h of stimulation, HNEPCs were collected for protein extraction. We examined the protein production of mTOR/p70S6K1 signaling pathway and proliferation molecules by WB. The results indicated that protein expression level the of p-mTOR, p-p70S6K1, P63, Ki67, cyclin E1, and CDK2 were increased in the IL-13 group and have statistical meaning (Figure 4A,C,E–I, *p* < 0.05), mTOR and p70S6K1 in HNESPCs were not over-expressed after the stimulation of IL-13, but have an increasing trend (Figure 4A,B, and D, *p* > 0.05). On the other hand, when we block mTOR (IL-13+rapamycin group) or S6K1 (IL-13+PF-4708671 group), protein production of p-mTOR, p-p70S6K1, Ki67, P63, cyclin E1, and CDK2 was decreased compared with IL-13 group (Figure 4, *p* < 0.05). As shown in Figure 4J,K, the ratio of p-mTOR/mTOR and p-p70S6K1/p-p70S6K1 is significantly increased in the IL-13 treatment group, consistent with the trend observed in tissue samples. This result suggests that IL-13 stimulation induces greater phosphorylation of p-mTOR and p-p70S6K1 proteins and promotes the activation of the mTOR pathway.

To detect HNESPCs proliferative ratio, portion stem/progenitor cells were made into cytospin slides for IF staining after stimulation, we found that the positive cells ratio of P63, Ki67, and double staining positive cells were increased in IL-13 group, but decreased in IL-13 + rapamycin group and IL-13 + PF-4708671 group (Figure 4L–O), vehicle control, P63: 0.35 ± 0.24, Ki67: 0.19 ± 0.13, double staining: 0.34 ± 0.13; IL-13, P63: 0.68 ± 0.10, Ki67: 0.48 ± 0.12, double staining: 0.53 ± 0.13; IL-13 + rapamycin, P63: 0.31 ± 0.12, Ki67: 0.04 ± 0.03, double staining: 0.32 ± 0.13; IL-13+PF-4708671, P63: 0.38 ± 0.12, Ki67: 0.19 ± 0.07, double staining: 0.06 ± 0.04.). In addition, we conducted EdU proliferation assays in HNESPCs and similarly observed that the ratio of positive cells in the IL-13 treatment group was significantly higher than in the other groups, thereby validating the previous experimental results, as shown in Figure S3.

Above all, the restriction of rapamycin caused low expression of p70S6K1 and p-p70S6K1, resulting in Ki67, CDK2, and cyclin E1 decreased, and the restriction of S6K1 also leading to downregulation of Ki67, CDK2, and cyclin E1. These results may elucidate our hypothesis that IL-13 could prompt the proliferative ability of nasal epithelial cells and that IL-13 works through mTOR/p70S6K1 pathway.

3. IL-13 may not annul its influence until HNESPCs complete differentiation in the ALI system.

To detect the IL-13 stimulatory effects on nasal epithelial cell differentiation. We separately treated the ALI system with IL-13 in 3 ways, the first group stimulated by PBS as a negative control was marked as “a”, the second groups just treated by IL-13 in the expansion phase were described as “b”, and the last group was treated by IL-13 at expansion and differentiation periods were called “c” (Figure 1A). qRT-PCR was performed to detect the mRNA expression level of the mTOR, p70S6K, and proliferation-related molecules Ki67, CDK2, and cyclin E1. The results indicated that mRNA production of mTOR and p70S6K1 did not increase significantly in “b” and “c” groups (Figure 1B,C, *p* > 0.05). P63 and Ki67 mRNA levels in “b” and “c” groups were significantly higher compared with “a” group (Figure 1D,E, *p* < 0.05), but continuous IL-13 stimulation during the ALI differentiation phase cannot further upregulate P63 and Ki67 mRNA expression even though they have an increasing trend (“c” group vs “b” group, Figure 4D,E, *p* > 0.05). Compared with “a” group, after the stimulation of IL-13, cyclin E1 mRNA production was increased but had no statistical significance in group “b” and “c” (Figure 1F, *p* > 0.05), CDK2 mRNA expression was significantly higher in “c” group, comparing to “a” group (Figure 1G, *p* < 0.05).

Then WB was performed to examine mTOR/p70S6K pathway and proliferation-related molecular expression. The protein expression of p-mTOR, p70S6K1, p-p70S6K1, P63, Ki67, CDK2, and cyclin E1 was upregulated after IL-13 stimulation (“b” and “c” group vs “a” group), however, the protein expression in the proliferation phase with IL-13 treatment (“b” group) and the whole phase with IL-13 treatment (“c” group) have no statistical significance (Figure 1, *p* > 0.05). From Figure 1J, we can observe that the p-mTOR/mTOR index in “c” group is significantly higher than in “a” group and “b” group, with a gradual increase between them. This suggests that the accumulation of p-mTOR is a progressive process. In Figure 1K, the p-p70S6K1/p70S6K1 index shows a gradual increase across “a” group, “b” group, and “c”group, although without statistical significance, which is consistent with the protein expression changes in Figure 1I. To some extent, this supports the overall trend that IL-13 activates the mTOR pathway, leading to increased expression of p-mTOR and p-p70S6K1. The ALI system was embedded with paraffin after the differentiation finished, and double IF staining with P63 and Ki67 (Figure 1L) indicated that the positive cell number of P63 and Ki67 were increased in group “b” and “c”, but there was no statistical difference between this two groups (Figure 1, M: P63^+^: group “a”, 17.04 ± 12.37; group “b”, 29.13 ± 13.81; group “c”, 26.86 ± 8.84; N: Ki67^+^: group “a”, 6.54 ± 6.95; group “b”, 15.29 ± 9.95; group “c”, 14.43 ± 5.70; O: double positive ratio: group “a”, 0.14 ± 0.21; group “b”, 0.52 ± 0.10; group “c”, 0.56 ± 0.21. The separate photos of Figure 1J were shown in Figure S2).

Nasal epithelial cells include basal, cilia, columnar, and goblet cells. Compared to other cells, goblet cells and cilia cells are more easily observed through staining. Furthermore, the morphological changes in cilia cells are more indicative of cellular responses. Therefore, in this study, we performed IF on both goblet cells and cilia cells, using the morphological alterations of cilia cells as an indicator to assess the effects of IL-13 on cell differentiation. Studies have shown that the primary mucin secreted by human epithelial goblet cells is MUC5AC [24, 25], so MUC5AC is regarded as a marker for goblet cells. And *β*-tubulin is regarded as a marker for cilia cells according to studies [26]. We performed the double IF staining of MUC5AC and *β*-tubulin after the differentiation was finished. Compared with group “a”, the expression level of *β*-tubulin in group “b” and group “c” were increased, but there was no statistical difference (Figure S1B); MUC5AC expression level in group “c” was significantly increased compared to group “a” and “b” (Figure S1A). Then we focus on the expression pattern of cilia cell in three groups, we could see that all cilia in group “a” were normal and well-shaped, but this phenomenon changed in group “b” and group “c”, the cilia cell expression pattern in group “b” was disordered and messy, although all cilia still located in the top area of epithelial cells. In group “c”, the cilia were much worse than in groups “a” and “b”; cilia were not just in the top but also the middle area of epithelial cells, exhibiting lodging, and the structure of cilia was abnormal. MUC5AC in group “b” was decreased compared with group “a” and “c”, but was just over-expressed in group “c” (Figure S1C).

## 4. Discussion

CRSwNP is one chronic inflammatory disease with several inflammatory cytokines acting on the nasal mucosa. IL-13 is the most represented cytokine in Th2 inflammation, which has been widely studied. Although other studies have shown that respiratory epithelial cells could secrete IL-13, but mRNA or protein expression of IL-13 cannot be detected in nasal epithelial cells treated or not treated with IL-13 in our previous studies and this study [11, 27]. Overexpressed IL-13 may be secreted from paracrine sources such as Th2 cells, ILC2s, and mast cells to induce nasal epithelium proliferation, implying that when the nasal mucosa is removed from the chronic inflammatory environment, it may reduce the abnormal proliferation of the self-repairment of nasal epithelium and ultimately reduce the formation possibility of nasal polyps. Therefore, nasal irrigation can reduce the inflammatory cytokines of nasal epithelium, reducing mucosal irritation, ameliorating the degree of mucosal edema, and maintaining the nasal environment [28].

In this study, we detected the expression level of IL-13, the initiating factors that overexpressed in CRSwNP, and explored its downstreaming factor mTOR/p70S6K1 pathway-related molecules, found that mTOR/p70S6K1 pathway exerts its physiological function mainly through post-translational phosphorylation of proteins because p-mTOR and p-p70S6K1 expression levels are clearly upregulated compared to mTOR and p70S6K1. mTOR could directly regulate downstream factors through post-translational phosphorylation and link with various proteins to form complexes, including mTOR complex 1 (mTORC1) and mTOR complex 2 (mTORC2). This study focused on mTORC1, which could induce cell anabolism and inhibit catabolism, promoting cell growth by regulating the cell cycle [29]. p70S6K1, one serine/threonine protein kinase, is the most widely studied type in the S6K family. It could promote protein synthesis and mRNA splicing, inhibit apoptosis, and participate in cytoskeleton synthesis. p70S6K1 can be found in the cytoplasm throughout the cell cycle. However, its nuclear localization is mainly in the G1 phase and depends on mTOR phosphorylation [30], in this study, we did not explore the nuclear localization of p70S6K1, but the expression after IL-13 stimulation in HNESPCs was upregulated, caused the proliferation activity enhanced, but rapamycin, an mTOR inhibitor, and PF-4708671, a p70S6K1 inhibitor, could restrict this phenomenon. p70S6K1, as a downstream effector of mTOR, promotes the G1/S phase transition during the cell cycle. CDK2 and cyclin E1 are key drivers of the G1-to-S phase progression. Therefore, mTOR indirectly facilitates G1/S transition through this regulatory cascade. Some research pointed out that IL-13 could influence the epithelial cells' proliferation in eosinophilic esophagitis, tumor cells [31, 32], but in CRSwNP, the function of IL-13 in proliferation activity is not explored enough. Our research group found that IL-13 could induce MUC5AC and ciliary changes, but did not mention the proliferation either. So we try to explain that type II inflammatory cytokine IL-13 may affect nasal epithelial basal cells proliferation.

**Figure 5:**
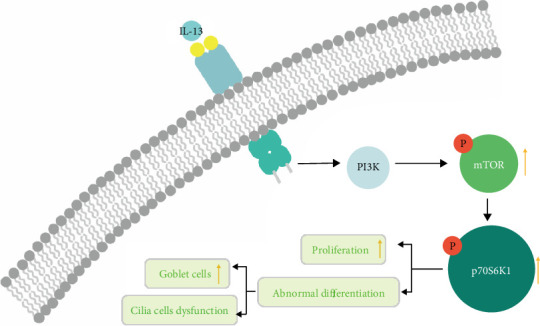
A schematic diagram demonstrating IL-13 may affect the proliferation and differentiation of nasal epithelial cells through the mTOR/p70S6K1 pathway.

To verify the function of IL-13 in nasal epithelial basal cells differentiation, the isolated HNESPCs were implanted in transwell chambers until differentiated into multilayer nasal epithelial cells, similar to the human nasal epithelial cells [33]. IL-13 was added into the medium during the expansion period of the ALI system, and cells were detected when the differentiation was finished. We found that the protein expressions of p-mTOR, p-p70S6K1, P63, Ki67, cyclin E1, and CDK2 were upregulated compared with the negative control group. The stimulatory effect of IL-13 on the mTOR/p70S6K1 pathway persists even after IL-13 withdrawal and consist until the differentiation of nasal epithelial cells is completed. In the differentiation phase, IL-13 stimulation can further improve the protein expressions of p-mTOR, p-p70S6K1, P63, cyclin E1, and CDK2, but the effect was not noticeable. In addition, we found that the positive cell counts of P63 and Ki67 were decreased with IL-13 stimulation in the whole phase compared to IL-13 stimulation in the proliferation phase of the ALI system, but without statistical significance, showing that maybe the IL-13 function in nasal epithelial basal cells were suspended, our research group have proved this phenomenon [10] as shown in Figure 5. In the IF staining results, we found that the cilia cells' expression pattern was changed, it's expression pattern in groups “b” (IL-13 in expansion phase) and “c” (IL-13 in expansion and differentiation phase) was much worse than in group “a” (negative control), this may indicate that IL-13 could persist it's influences even if we revoke the IL-13 stimulation during HNESPC differentiation period. Milad et al. pointed out that an mTOR-related signaling pathway exists in multiple complex negative feedback regulations [34]. When mTOR is overexpressed, negative feedback will be activated to inhibit the expression of mTOR and its downstream factor p70S6K1, thus reducing the expression of cell proliferation-related factors. IL-13 stimulation could persist in the ALI system's whole phase, and extending the stimulation duration may cause negative feedback activation, which may be why the number of P63 positive cells, Ki67 protein expression, and Ki67 positive cells decreased compared with IL-13 stimulation only during the amplification phase.

These results indicate that the excitatory effect of IL-13 on the mTOR/p70S6K1 pathway may mainly act on the proliferative and differential phase of the nasal epithelium. Activate mTOR/p70S6K1 signaling pathway and the expression of related proliferative indicators can be increased to accelerate the cell cycle transformation, and finally, the abnormal proliferation and differentiation of nasal epithelium may be generated. These may be associated with the development of nasal polyps. But in this study, we just observed that stimulation of IL-13 in the expansion phase also accelerated cilia cell dysfunction; the mechanism was not explored enough. We will continue to focus on the IL-13 function in nasal epithelial basal cells, trying to explain why short IL-13 stimulation could affect cilia cells.

## 5. Conclusion

In this study, the expression of mTOR/p70S6K1 pathway-related molecules, basal cell marker P63, cell proliferation marker Ki67, and cell cycle-related factors cyclin E1, CDK2 were upregulated after IL-13 stimulation of nasal epithelial cells including HNESPCs, the distribution pattern of cilia cell was disordered and messy, which was similar to the expression pattern in CRSwNP tissues. Therefore, we hypothesize that IL-13 leads to the phosphorylation of related molecules of the mTOR/p70S6K1 pathway, promoting the proliferation and differentiation of the nasal epithelium, which leads to the generation of nasal polyps.

## Figures and Tables

**Table 1:** Patients' basic characteristics.

	CRSwNP	HC
Sample size	65	26
Gender (male/female)	53/12	22/4
Age (years)	(43.29 ± 19.44)	(33.50 ± 12.54)*⁣*^*∗*^
Smoking (smoker/nonsmoker)	19/46	11/25
Asthma (yes/no)	8/57	0/26
Allergic (yes/no)	17/48	5/21
First diagnose of CRSwNP	31/48	—
Recurrent CRSwNP	17/48	—

*Note*: Student's *t*-test was performed to analyze the significance of ages (P), other categorical variables were used *χ*^2^ test. (mean ± SD).

Abbreviations: CRSwNP, chronic rhinosinusitis with nasal polyps; HC, healthy control.

*⁣* ^*∗*^means *p* < 0.05.

**Table 2:** Primers sequences of reverse transcription-polymerase chain reaction.

Gene	Toward	Sequences
GAPDH	Forward	ACAGTTGCCATGTAGACC
	Reverse	TTTTTGGTTGAGCACAGG

mTOR	Forward	GCAGATTTGCCAACTATCTTCGG
	Reverse	CAGCGGTAAAAGTGTCCCCTG

p70S6K1	Forward	TTTGAGCTACTTCGGGTACTTGG
	Reverse	CGATGAAGGGATGCTTTACTTCC

P63	Forward	CCACCTGGACGTATTCCACTG
	Reverse	TCGAATCAAATGACTAGGAGGGG

Ki67	Forward	ACGCCTGGTTACTATCAAAAGG
	Reverse	CAGACCCATTTACTTGTGTTGGA

Cyclin E1	Forward	AAGGAGCGGGACACCATGA
	Reverse	ACGGTCACGTTTGCCTTCC

CDK2	Forward	TGTTTAACGACTTTGGACCGC
	Reverse	CCATCTCCTCTATGACTGACAGC

## Data Availability

All data generated or analyzed during this study are included in this published article and its supporting information files.

## Nomenclature

CRSwNP: chronic rhinosinusitis with nasal polyps
ILC2s: group 2 innate lymphoid cells
mTOR: mammalian target of rapamycin
p70S6K1: Ribosomal protein S6 kinase 1
CDK2: cyclin-dependent kinase 2
HC: healthy control
ESS: endoscopic sinus surgery
HNESPCs: human nasal epithelial stem/progenitor cells
ALI: air-liquid interface
RT-PCR: reverse transcription-polymerase chain reaction
WB: western blot
IHC: immunohistochemistry
IF: immunofluorescent.

## Supporting Information

Supporting Information Figure S1: Representative images of ALI system with MUC5AC (ab3649, 1:500) and β-tubulin (ab179513,1:500) double IF staining were photographed with the confocal laser scanning microscope. Figure S2: Representative images of ALI system with P63 and KI67 double IF staining were photographed with the confocal laser scanning microscope. Figure S3: Represented images of EdU staining were photographed with fluorescence microscopy and the ratio of EdU-positive cells in HNEPCs. Figure S4: Characterization of HNESPCs. A–B, illustrate the growth changes of stem cells from Day 3 to Day 6. 200x magnification, scale bar = 100 µm. The area within the red box represents the stem cells. E-H, display the immunofluorescence staining of KRT5+/P63+ cells. KRT-5+/P63+ cells were considered as stem/progenitor cells. 400x magnification, scale bar = 50 µm. Table S1: Antibodies for staining and western blot. Table S2: *p* value of Figure 1. Table S3: *p*-value of Figure 2. Table S4: *p*-value of Figure 3. Table S5: *p*-value of Figure 4.
