# Supplementary material for: IL-13 May Could Enhance the Proliferation and Affect the Differentiation of Nasal Epithelium Basal Cells Through the mTOR/p70S6K1 Pathway in Chronic Rhinosinusitis With Nasal Polyps
Source: Mediators Inflamm. 2025 May 21;2025:8108993. doi: 10.1155/mi/8108993 (PMC12119155; doi:10.1155/mi/8108993)
Supplement: Supporting Information — Figure S1: Representative images of ALI system with MUC5AC (ab3649, 1:500) and β-tubulin (ab179513,1:500) double IF staining were photographed with the confocal laser scanning microscope. Figure S2: Representative images of ALI system with P63 and KI67 double IF staining were photographed with the confocal laser scanning microscope. Figure S3: Represented images of EdU staining were photographed with fluorescence microscopy and the ratio of EdU-positive cells in HNEPCs. Figure S4: Characterization of HNESPCs. A–B, illustrate the growth changes of stem cells from Day 3 to Day 6. 200x magnification, scale bar = 100 µm. The area within the red box represents the stem cells. E-H, display the immunofluorescence staining of KRT5+/P63+ cells. KRT-5+/P63+ cells were considered as stem/progenitor cells. 400x magnification, scale bar = 50 µm. Table S1: Antibodies for staining and western blot. Table S2: p value of Figure 1. Table S3: p-value of Figure 2. Table S4: p-value of Figure 3. Table S5: p-value of Figure 4. [file 8108993.f1.docx]

Supplementary Information

| Table S1. Antibodies for staining and western blot | | | | |
| --- | --- | --- | --- | --- |
| Antibody | Dilution | | Catalog number | Company |
|  | Staining | Western |  |  |
| β-Actin | 1:50 | 1:2000 | TA-09 | ZSGB-BIO |
| mTOR | 1:400 | 1:6000 | ab32028 | Abcam |
| p-mTOR | 1:50 | 1:1000 | ab131538 | Abcam |
| P70S6K1 | 1:50 | 1:1000 | #9202 | Cell signaling technology |
| p-p70S6K1 |  | 1:1000 | #97596 | Abcam |
| P63 | 1:100 | 1:1000 | ab124762 | Abcam |
| Ki67 | 1:250 | 1:2000 | ab16667 | Abcam |
| Ki67 | 1:100 |  | ab279653 | Abcam |
| cyclin E1 |  | 1:1000 | ab3927 | Abcam |
| CDK2 |  | 1:1000 | #18048S | Cell signaling technology |
| MUC5AC | 1:500 |  | ab3649 | Abcam |
| β-tubulin | 1:500 |  | ab179513 | Abcam |

|  | Table S2. P value of figure 1 | | | | |  |
| --- | --- | --- | --- | --- | --- | --- |
|  | Fig 1A-D | Fig 1E | Fig 1G | Fig 1H | Fig 1I | Fig 1K |
| IL-13 | <0.0001 |  |  |  |  |  |
| mTOR | 0.0005 | 0.0135 | >0.9999 |  |  | 0.0539 |
| p-mTOR |  |  | 0.0080 |  |  | 0.0458 |
| p70S6K1 | <0.0001 | <0.0001 | >0.9999 |  |  | 0.0653 |
| p-p70S6K1 |  |  | 0.0172 |  |  | 0.0328 |
| P63 | <0.0001 |  | 0.0164 |  |  | 0.0379 |
| p-mTOR/ mTOR |  |  |  | 0.0366 |  |  |
| p-p70S6K1/ p70S6K1 |  |  |  |  | 0.0386 |  |

| Table S3. P value of figure 2 | | | |
| --- | --- | --- | --- |
|  | Fig 2A | Fig 2 B-D | Fig 2E |
| Ki67 | 0.0075 | <0.0001 | 0.0270 |
| CDK2 | 0.0228 | 0.0306 |  |
| cyclinE1 | 0.0420 | 0.0310 |  |

| Table S4 P value of figure 3 | | | | | | | |
| --- | --- | --- | --- | --- | --- | --- | --- |
|  | Fig 3B-K | | | Fig 3M-O | | | |
|  | (1) vs. (2) | (2) vs. (3) | (2) vs. (4) | (1) vs. (2) | (2) vs. (3) | | (2) vs. (4) |
| mTOR | >0.9999 | 0.3467 | >0.9999 |  |  |  | |
| p-mTOR | 0.0313 | 0.0096 | 0.0141 |  |  |  | |
| p70S6K1 | 0.4596 | 0.4868 | 0.0941 |  |  |  | |
| p-p70S6K1 | 0.0360 | 0.0478 | 0.0036 |  |  |  | |
| P63 | 0.0431 | 0.0094 | 0.0108 | 0.0171 | 0.0250 | 0.0044 | |
| Ki67 | 0.0054 | 0.0030 | <0.0001 | 0.0019 | 0.0018 | <0.0001 | |
| P63^+^/Ki67^+^ |  |  |  | 0.0388 | 0.0341 | <0.0001 | |
| CDK2 | 0.0345 | 0.0002 | <0.0001 |  |  |  | |
| cyclinE1 | 0.0186 | 0.0035 | 0.0003 |  |  |  | |
| p-mTOR/ mTOR | 0.0030 | 0.0421 | 0.0242 |  |  |  | |
| p-p70S6K1/ p70S6K1 | 0.0250 | 0.0370 | 0.0263 |  |  |  | |
| (1) cells were pretreated with DMSO (0.1%) as the vehicle control for 1 hour; (2) cells were treated with IL-13 (10 ng/ml) only; (3) cells were pretreated with Rapamycin (10 nM, Selleck, Houston, Texas, USA), a specific mTOR inhibitor, for 1 hour; (4) cells were pretreated with PF-4708671 (10 nM, Selleck, Houston, Texas, USA), a specific p70S6K1 inhibitor, for 1 hour. | | | | | | | |

| Table S5. P value of figure 4 | | | | | | | | | |
| --- | --- | --- | --- | --- | --- | --- | --- | --- | --- |
|  | Fig 4B-G | | | Fig 4I-K | | | Fig 4M-O | | |
|  | a vs. b | a vs. c | b vs. c | a vs. b | a vs. c | b vs. c | a vs. b | a vs. c | b vs. c |
| mTOR | 0.6210 | 0.2080 | 0.1531 | 0.2531 | 0.9670 | 0.1568 |  |  |  |
| p-mTOR |  |  |  | 0.0179 | 0.0035 | >0.9999 |  |  |  |
| p70S6K1 | 0.4146 | >0.9999 | 0.0951 | 0.0235 | 0.0176 | 0.2318 |  |  |  |
| p-p70S6K1 |  |  |  | 0.0179 | 0.0373 | >0.9999 |  |  |  |
| P63 | 0.0081 | 0.0081 | >0.9999 | 0.0044 | 0.0044 | >0.9999 | 0.0019 | 0.0184 | 0.8025 |
| Ki67 | 0.0130 | 0.0223 | >0.9999 | 0.0024 | 0.0219 | >0.9999 | 0.0019 | 0.0046 | >0.9999 |
| P63^+^/Ki67^+^ |  |  |  |  |  |  | 0.0124 | 0.0089 | 0.6977 |
| CDK2 | >0.9999 | 0.0226 | 0.0975 | 0.0216 | 0.0080 | >0.9999 |  |  |  |
| p-mTOR/ mTOR |  |  |  | >0.9999 | 0.0285 | 0.2515 |  |  |  |
| p-p70S6K1/ p70S6K1 |  |  |  | 0.0806 | 0.3415 | >0.9999 |  |  |  |
| cyclinE1 | 0.7231 | 0.2377 | 0.6248 | 0.0110 | 0.0417 | >0.9999 |  |  |  |
| a: added PBS as vehicle control; b: added IL-13 (10ng/ml) during the duration of proliferation; c: added IL-13 (10ng/ml) during the entire duration of proliferation and differentiation | | | | | | | | | |


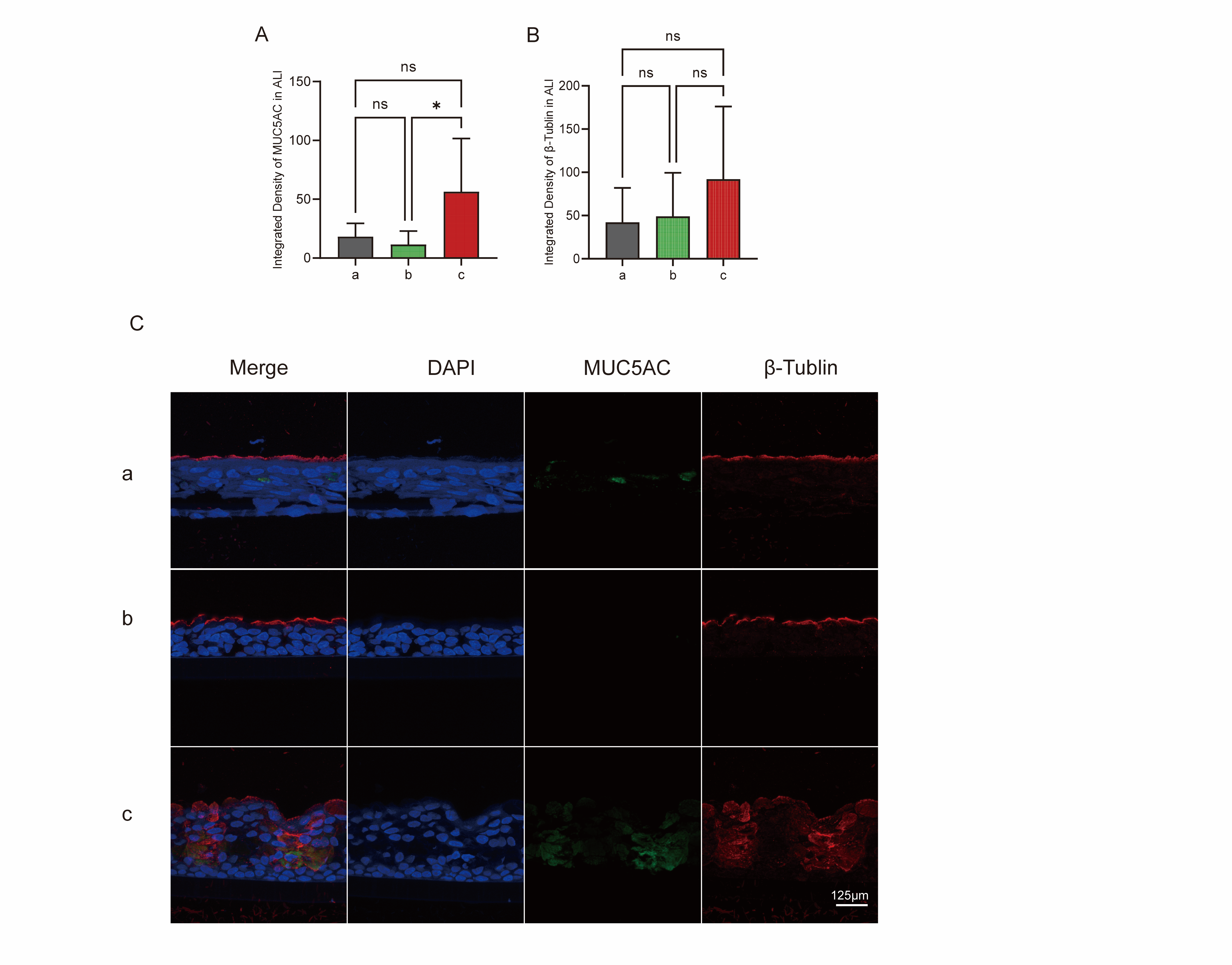


**Figure S1.** **Representative images of ALI system with MUC5AC (ab3649, 1:500) and β-tubulin (ab179513,1:500) double IF staining were photographed with the confocal laser scanning microscope.** a, PBS as vehicle control; (b) IL-13 (10ng/ml) added in the proliferation phase; (c) IL-13 (10ng/ml) added in both of proliferation and differentiation phases. ×1000 magnification, scale bar = 125 µm. ALI, air-liquid interface.


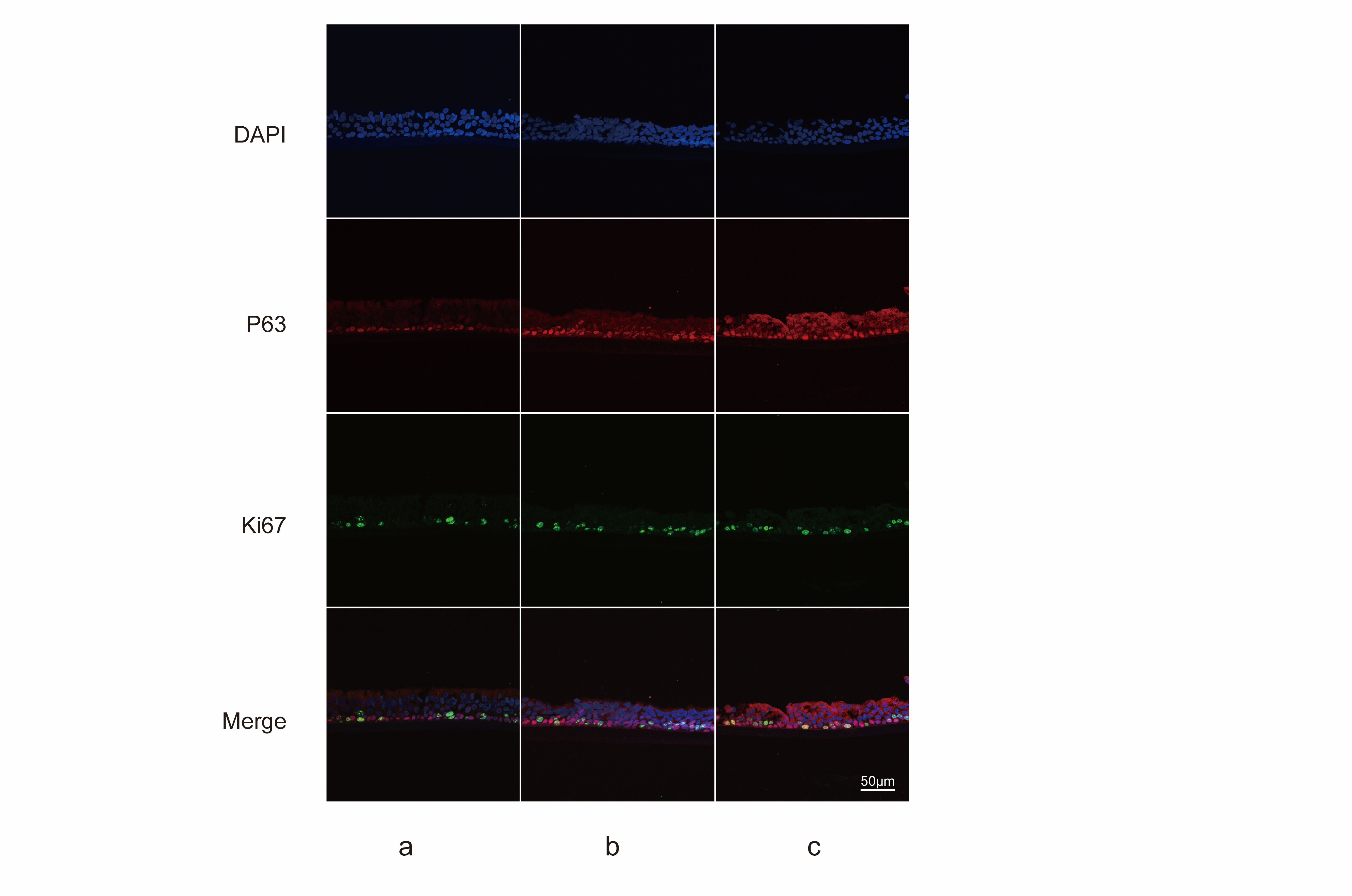


**Figure S2. Representative images of ALI system with P63 and KI67 double IF staining were photographed with the confocal laser scanning microscope.** a, PBS as vehicle control; (b) IL-13 (10ng/ml) added in the proliferation phase; (c) IL-13 (10ng/ml) added in both of proliferation and differentiation phases. ×400 amplification, scale bar = 50µm. ALI, air-liquid interface.

**
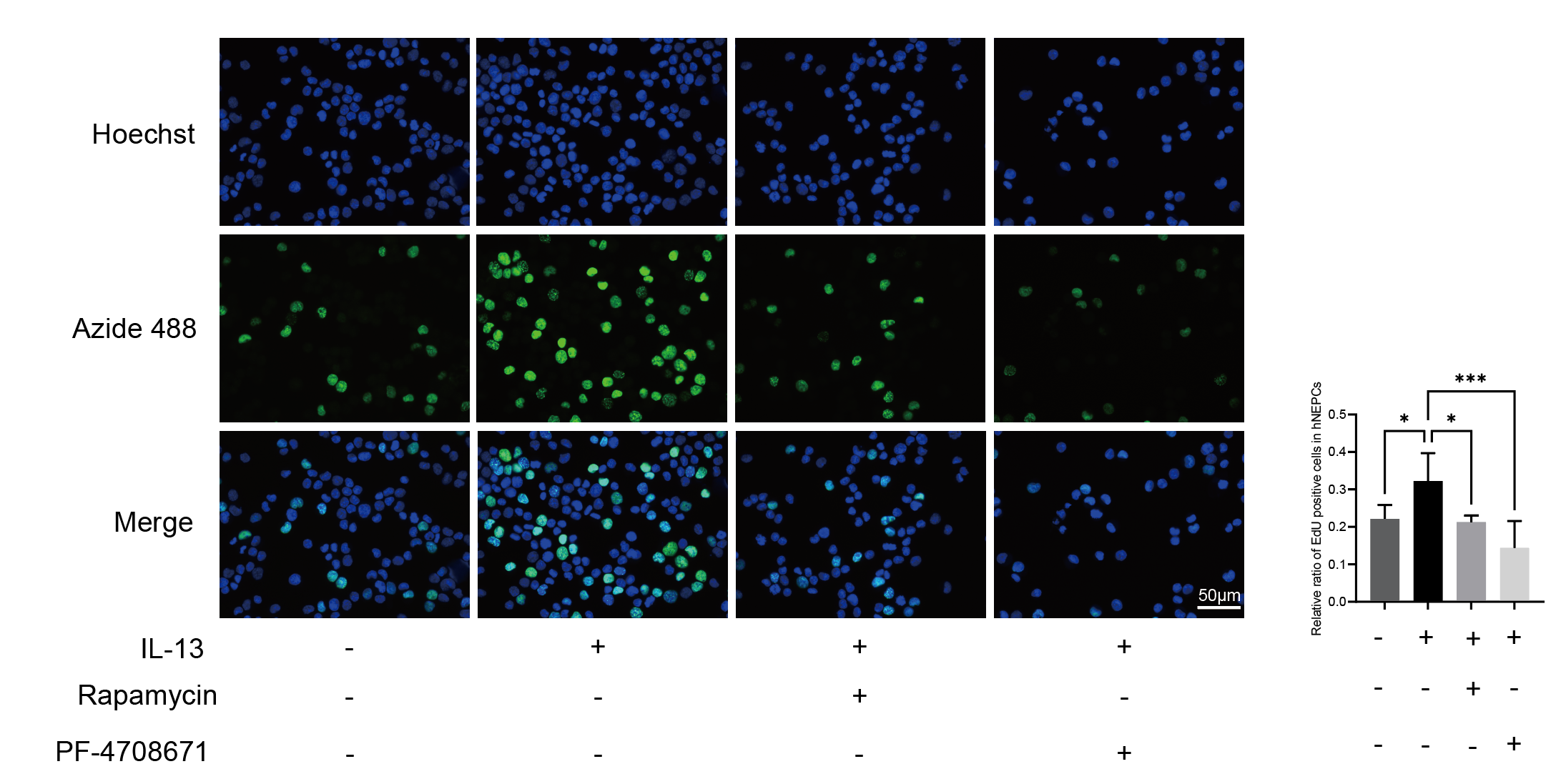
**

**Figure S3.** **Representative images of EdU staining were photographed with fluorescence microscopy and the ratio of EdU-positive cells in HNESPCs,** ×400 magnification, scale bar = 50µm. Data were shown as mean±SD, statistical significance was analyzed by One-Way ANOVA test. HNESPCs, Human nasal epithelial stem/progenitor cells; * P<0.05; ** P<0.01; *** P<0.001.


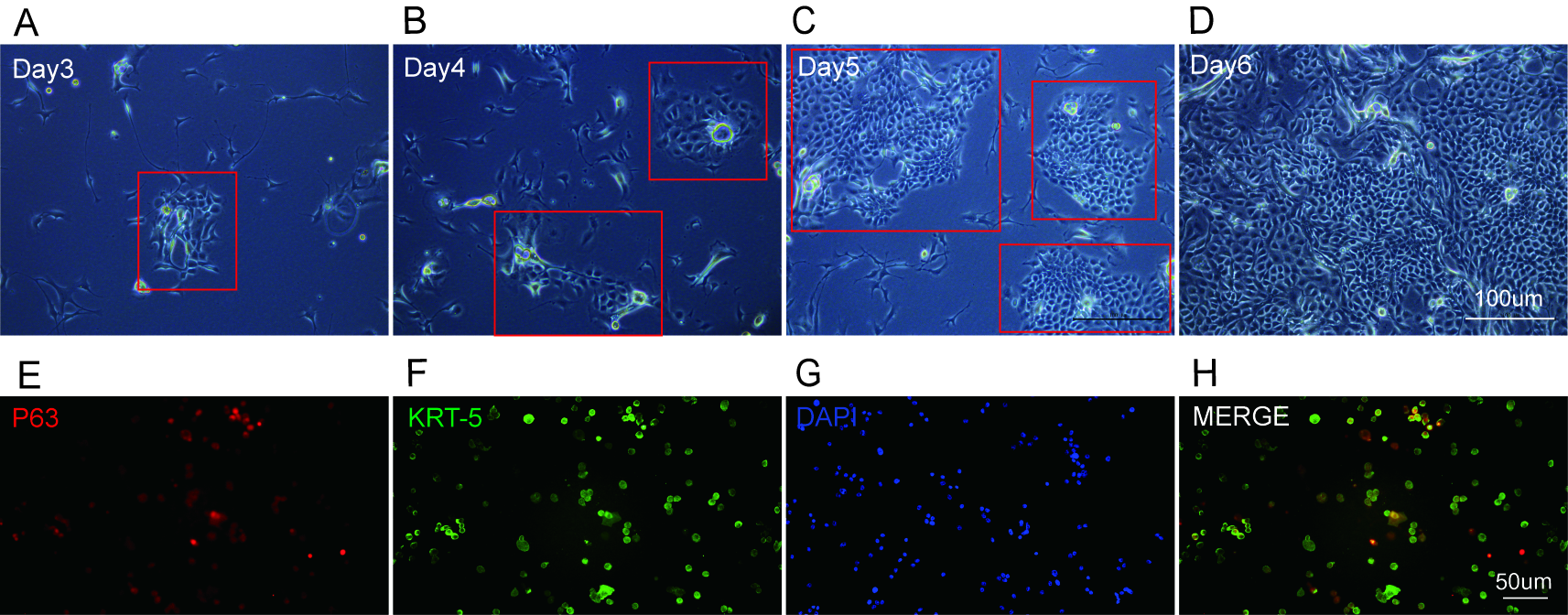


**Figure S4. Characterization of HNESPCs.** A-D, illustrate the growth changes of stem cells from Day 3 to Day 6. ×200 magnification, scale bar = 100µm. The area within the red box represents the stem cells. E-H, display the immunofluorescence staining of KRT5+/P63+ cells. KRT-5+/P63+ cells were considered as stem/progenitor cells. ×400 magnification, scale bar = 50µm.
